# Peanut shell-derived activated carbon incorporated with ZnCo_2_O_4_ in alkaline solution for high performance asymmetric supercapacitors

**DOI:** 10.1039/d6ra04675d

**Published:** 2026-09-03

**Authors:** Jing-Song Lv, Cong Lei, Waseem Abbas, Changlin Dong, Muhammad Ehsan Mazhar, Imran Shakir, M. Naziruddin Khan, Rehana Bibi, Shakeel Abbas, Muhammad Usman Yousaf, Areeba

**Affiliations:** a Analytical and Testing Center, Guizhou University of Engineering Science Guizhou 551700 China songlvj@gmail.com; b School of Mechanical Engineering, Guizhou University of Engineering Science Guizhou 551700 China; c Institute of Physics, Bahauddin Zakariya University Multan 60800 Pakistan Dr.waseemabbas@bzu.edu.pk; d School of Material Science and Engineering, Shanghai Jiao Tong University Shanghai 200240 China; e Department of Physics, Faculty of Science, Islamic University of Madinah Madinah 42351 Saudi Arabia; f Department of Chemistry, Women University Swabi Pakistan; g Department of Chemistry, Government College University Faisalabad Pakistan

## Abstract

Supercapacitors have attracted a lot of interest in energy storage technology because of their affordability, large capacity, high energy efficiency, and exceptional stability. In this work, zinc cobaltite (ZnCo_2_O_4_) was combined with a biomass-derived carbon material derived from peanut shells to create a ZnCo_2_O_4_/PAC nanocomposite using an easy and affordable hydrothermal process. The combination of ZnCo_2_O_4_ and Powder Activated Carbon (PAC) forms a good conductive network that provides extra redox-active sites and facilitates electron transit. When compared to pristine ZnCo_2_O_4_ (530 F g^−1^), the designed electrode had the most effective charge storage behavior in basic electrolyte, attaining a capacitance of 781 F g^−1^ at 1 A g^−1^. Besides, an asymmetric supercapacitor (ZnCo_2_O_4_/PAC//AC) device presented a notable capacitance of 160 F g^−1^ with an excellent energy density of 50 Wh kg^−1^. The device retained 92% of its initial capacitance retention after 6000 cycles, showing its good cycling reliability. The remarkable performance is attributed to ZnCo_2_O_4_'s improved charge transfer, and PAC's high surface area, which overall improved the electronic conductivity and ion diffusion. These outcomes reveal that the ZnCo_2_O_4_/PAC composite provides directions for the development of future high-performance supercapacitors.

## Abstract 2

Peanut shell-derived PAC enables a porous ZnCo_2_O_4_/PAC hybrid with enhanced active sites, conductivity and ion diffusion. The ZnCo_2_O_4_/PAC//AC asymmetric supercapacitor delivers 50 Wh kg^−1^ energy density and 92% retention after 6000 cycles.

## Graphical abstract

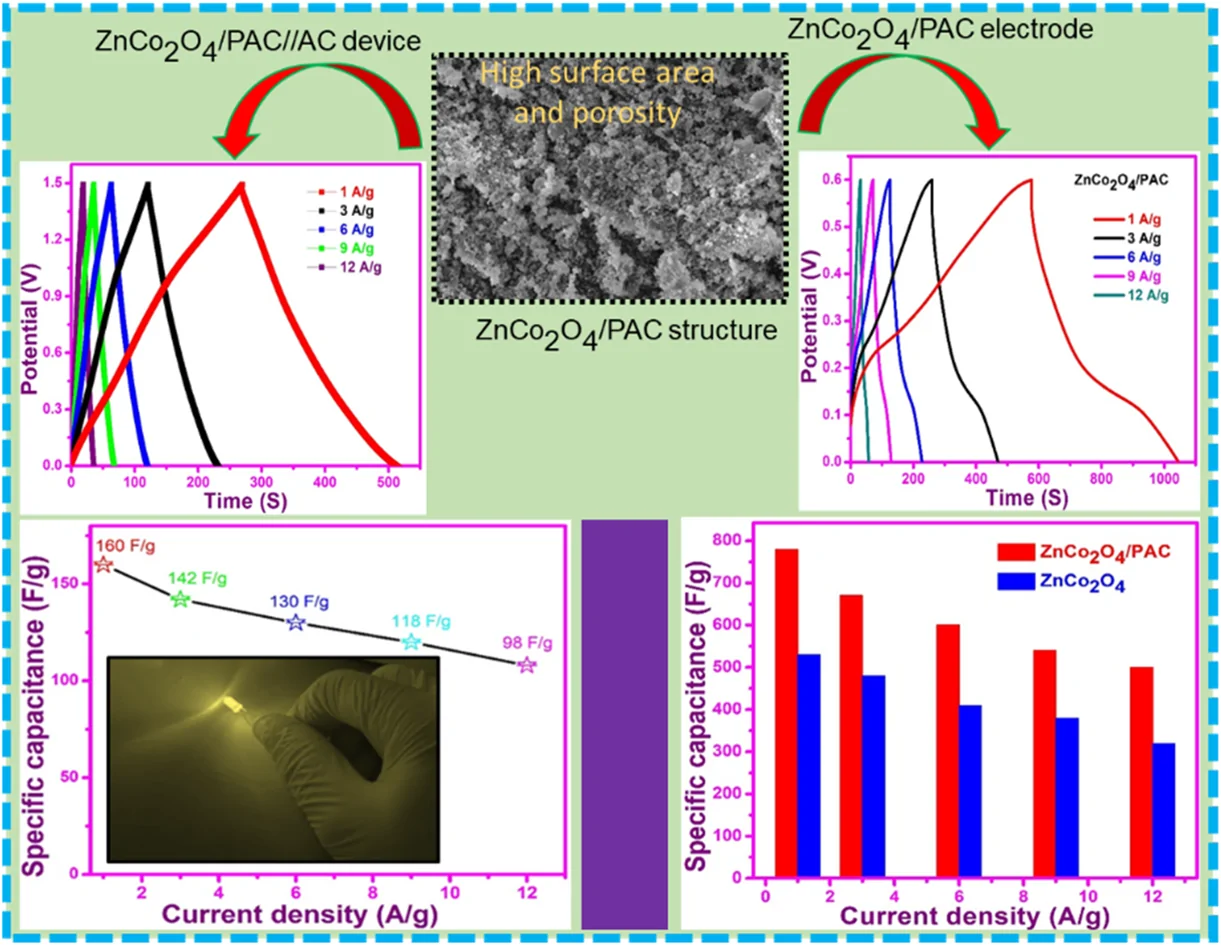

## 1. Introduction

To fulfil the rapidly increasing global energy needs, it is crucial to investigate cutting-edge renewable energy resources and storage methods. Thus, renewable energy sources are becoming more prevalent worldwide. This basically explains why sustainable energy production and energy storage systems are necessary. Scientists are working to create gadgets based on renewable materials to reduce environmental pollution, mitigate global warming, and preserve economic equilibrium for future generations. Because of their exceptional power density, quick charging and discharging, and exceptional cycling stability, supercapacitors have emerged as a major topic of research.^1^ Supercapacitors are generally grouped according to the methods they use to store electrical energy. By building up charges, electrochemical double-layer capacitors (EDLCs) store energy physically at the boundary shared by the electrolyte and electrode. Conversely, pseudocapacitors depend upon fast and reversible faradaic oxidation-reduction reactions at the electrode surface rather than a purely electrostatic charge mechanism. This mechanism provides a greater charge-storage capacity, making pseudocapacitors promising candidates for high performance energy-storage systems.^2,3^ However, the low specific capacitance and poor cyclic stability of supercapacitors restrict their applications for real world applications. Researchers are working to develop innovative electrode materials to improve the electrochemical effectiveness of supercapacitors in order to address these problems.^4^ For supercapacitor electrodes, transition metal oxides (TMOs) have become quite appealing, thanks to their exceptional specific capacity, superior electrical conductivity, and distinctive crystalline architectures.^5–7^ Among these, ZnCo_2_O_4_ has emerged as a promising electrode material for energy storage systems, owing to its multiple valence states, rich redox chemistry, and intrinsically low ion diffusion resistance, which collectively enhance the electrochemical charge storage performance.^8,9^ Despite its promising electrochemical characteristics, ZnCo_2_O_4_ suffers from several intrinsic drawbacks that limit its practical utilization, including low electrochemically accessible surface area, slow charge-transfer kinetics and structural degradation during repeated charging/discharging cycles. Doping with other transition metals, incorporation of carbon-based materials (derived from biomass), surface-controlled morphology,^10,11^ and defect engineering are some of the strategies used to overcome these obstacles.^12^ Biomass-derived carbon is considered a low-cost, efficient electrode material for SCs due to its important features including high electrical conductivity, better pore size distribution, and exceptional chemical durability. Additionally, use of biomass derivatives contributes to efficient sustainable energy development and environmental protection. Numerous energy and environmental applications use biomass carbon sources such as peanut shells (PS), almond shells, coconut shells, tea leaves, poplar wood, and others. Because PAC's larger surface area makes it easier to store charges, it has attracted a lot of interest from energy storage applications like batteries and SCs. Powder Activated Carbon (PAC) has drawn a lot of attention to formulate high performance electrodes for SCs, because of their inexpensive, environmentally friendly and high porous structure. Additionally, PAC offer many electrochemical active sites for the high-performance energy storage applications.^13^ The PAC appears as thicker, rough patches integrated into the ZnCo_2_O_4_ due to its substantial surface area and interconnected permeable network, which enhances electrical conductivity and active sites.^14^

It is observed that both ZnCo_2_O_4_ and PAC individually have some limitations like low energy density, low power density, poor cyclic stability. These limitations can be addressed by constructing ZnCo_2_O_4_/PAC composite. Their tunable microporous structure, customizable pore dimensions, and large surface areas make them ideal for energy storage devices.^15^ Zhang *et al.* derived activated carbon from potato and investigated their electrochemical performance capacitance and cyclic stability tested in a 6 M KOH aqueous electrolytic solution.^16^ Khadka *et al.* developed activated carbon from coffee waste and evaluated it as a supercapacitor electrode material, showing a high capacitance of 113.81 F g^−1^.^17^ Lee *et al.* fabricated of GnPs and attained specific capacitance value of 267 F g^−1^ with good cycling stability.^18^ Sharma *et al.* successfully synthesized g-C_3_N_4_@ZnCo_2_O_4_ composite-based electrode and attained maximal specific capacity of 157 mAh g^−1^ at 4 A g^−1^ with capacity retention of 71%.^19^ In contrast to literature,^16–19^ the current study explores the synergistic role of ZnCo_2_O_4_/PAC structure provides extraordinary morphological features of porous sponge-like morphology with large surface area.

In this work, ZnCo_2_O_4_/PAC composite was successfully prepared through an easy and affordable hydrothermal method. The prepared material was electrochemically evaluated as a supercapacitor electrode. The ZnCo_2_O_4_/PAC electrode exhibited remarkable charge-storage capability, carrying a good capacitance of 780 F g^−1^. Additionally, after 6000 charge–discharge cycles, the developed asymmetric supercapacitor device showed remarkable energy storage of 50 Wh kg^−1^ and maintained 92% capacitance retention. Significantly, these findings support the ZnCo_2_O_4_/PAC composite's potential for high-performance supercapacitor applications.

## 2. Experimental

### 2.1 Chemicals and materials

In this research work cobalt nitrate hexahydrate (Co(NO_3_)_2_·6H_2_O), ≥98%, Sigma-Aldrich) and zinc nitrate hexahydrate (Zn(NO_3_)_2_·6H_2_O), Sigma-Aldrich) are used for synthesis of nanoparticles of zinc cobalt oxide. Potassium hydroxide (KOH, ≥85%, Merck) used as precipitating agent and peanut shell carbon (C) is also incorporated in oxides of zinc cobalt to enhance the electrochemical reactivity.

### 2.2 Synthesis of activated carbon from peanut shell

After being washed with deionized water to remove contaminants, 10 g of peanut shells were heated to 300 °C for 5 hours in a muffle furnace. 1.0 M KOH was used to activate the sample. The carbonization process was then started by heating the sample to 800 °C in an argon atmosphere in a porcelain boat within a tube furnace. After removing the unreacted activating agent with 3 M HCl, the resulting carbon powder was sonicated for 10 hours and then cleaned with water. After filtration, the activated carbon powder (PAC) was dried for 2 hours at 200 °C in an oven.

### 2.3 Synthesis of zinc cobalt oxide

Zinc cobalt oxide (ZnCo_2_O_4_) was prepared using a hydrothermal synthesis method. Initially, cobalt nitrate (Co(NO_3_)_2_, 5.4 g) and zinc nitrate (Zn(NO_3_)_2_, 1.2 g) solutions were dissolved by magnetic stirrer in 30 mL of DI water, continuously to ensure full dissolution. Then potassium hydroxide KOH was added in the above solution drop by drop with the help of dropper and mixed for 30 minutes. The resulting solution was then placed in a 50 mL autoclave lined with Teflon and baked at 120 °C for six hours. Once the solution has cooled to room temperature, it is centrifuged until refined precipitates are obtained. Take off these precipitates into a petri dish and dried at 60 °C for 5 hours. The dried sample was then further annealed for four hours at 500 °C in a furnace.

### 2.4 Preparation of ZnCo_2_O_4_/PAC heterostructure

Sonication was used to prepare the ZnCo_2_O_4_/PAC heterostructure. The ZnCo_2_O_4_/PAC heterostructure was prepared by dispersing as-synthesized ZnCo_2_O_4_ (100 mg) and PAC (20 mg) in 15 mL of ethanol and sonicating the mixture in an ice bath for 3 hours to maintain stable temperature. The produced ZnCo_2_O_4_/PAC heterostructure was then employed for additional characterizations after being dried for 5 hours at 50 °C.

### 2.5 Electrodes fabrication

The metallic electrodes were made with 80 wt% active material (ZnCo_2_O_4_, and ZnCo_2_O_4_/PAC), 10 wt% PVDF and 10 wt% carbon black. Using *N*-methyl-2-pyrrolidone (NMP) as the solvent, the active material, conductive polyvinylidene fluoride (PVDF) and carbon black binder were thoroughly blended to produce the homogeneous slurry. To ensure a clean and chemically active surface, nickel foam was ultrasonically treated in dilute HCl solution, thoroughly cleaned with ethanol (C_2_H_5_OH), and subsequently dried at 60 °C overnight. The resulting slurry was drop-cast onto (1.0 cm × 2.0 cm) nickel foam and dried for 24 hours at 60 °C. The same procedure was used to manufacture the activated carbon (AC) electrode.

### 2.6 Characterization techniques

X-ray diffraction (XRD) was utilized to examine the crystallographic structures of the as-synthesized samples using X-ray with Cu Kα radiation (*λ* = 1.5406 Å). The diffraction profiles were recorded over a 2*θ* range of 10–80° to identify the crystalline phases and evaluate structural features. The average crystallite size of the samples was determined using the diffraction intensity peak. The produced materials' surface structure and microstructural characters were studied using field emission SEM with a TESCAN MIRA A-3 system. The surface chemical composition and electronic structure of the produced material were systematically characterized by X-ray photoelectron spectroscopy (XPS) using the SuperESCA beamline at Elettra Sincrotrone Trieste, Italy. The pore size distribution was determined from the N_2_ adsorption–desorption isotherms using the Barrett–Joyner–Halenda (BJH) method. To clarify the surface chemical functionalities and characteristic bonding environments of the synthesized samples were examined by Fourier transform infrared (FTIR) spectroscopy using a Nicolet iS50 spectrometer.

### 2.7 Electrochemical measurements

The electrochemical behaviour of the fabricated ZnCo_2_O_4_ and ZnCo_2_O_4_/PAC electrodes were evaluated using a CHI660D electrochemical workstation in a three-electrode system. The ZnCo_2_O_4_, and ZnCo_2_O_4_/PAC electrodes were used as working electrode, while a Pt plate and Hg/HgCl_2_ were employed as counter and reference electrode, respectively. Cyclic voltammetry (CV) was carried out at a scan rate ranging from 10 to 60 mV s^−1^, and galvanostatic charge–discharge (GCD) investigations were carried out at current densities between 1 and 12 A g^−1^. Electrochemical impedance spectroscopy (EIS) measurements were performed at room temperature over a frequency range of 10 mHz to 100 kHz. Additionally, a two-electrode preparation was used to evaluate the electrochemical performance of the fabricated ZnCo_2_O_4_/PAC//AC asymmetric supercapacitor.

The capacitance of the manufactured electrodes was ascertain using eqn (1);1
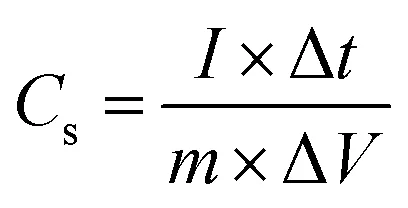


Additionally, eqn (2) and (3) were utilized to calculate the device's energy density and power density;2
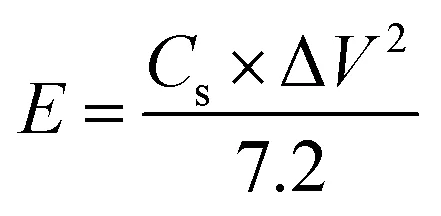
3
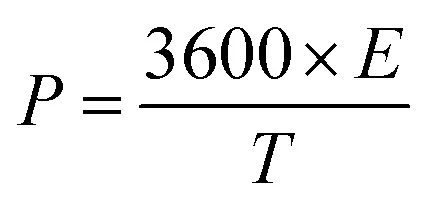


## 3. Results and discussion

Scheme 1 displays the schematic illustration of the synthesis steps of powder activated carbon (PAC) from waste peanut shells, synthesis of ZnCo_2_O_4_ structure and formation of ZnCo_2_O_4_/PAC hybrid structure.

**Scheme 1:**
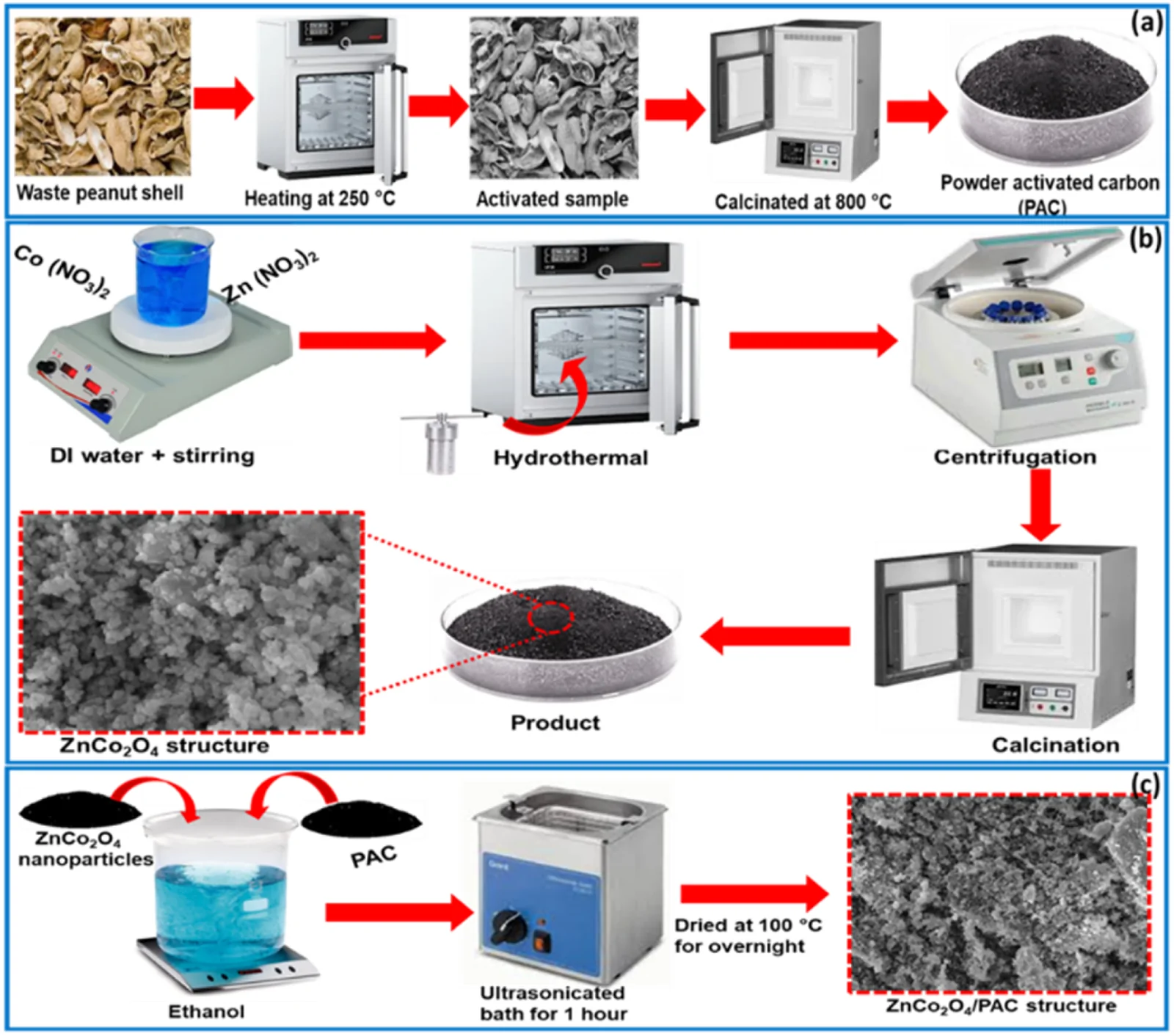
Schematic design of the synthesis steps: (a) preparation of powder activated carbon (PAC) from waste peanut shells (b) synthesis of ZnCo_2_O_4_ structure (c) formation of ZnCo_2_O_4_/PAC structure through ultrasonication.

### 3.1 Morphological and structural analysis

SEM was used to analyze the structure and morphology of synthesized PAC, ZnCo_2_O_4_ and ZnCo_2_O_4_/PAC samples. Fig. 1(a and b) illustrate the SEM photographs at different magnifications of ZnCo_2_O_4_ morphology, which reveals hierarchical microspheres constructed from self-assembled nanoparticles with limited visible porosity. Fig. 1(c and d) shows the 3D interconnected sponge-like morphology of ZnCo_2_O_4_/PAC which reveals rough, highly porous and agglomerated nanoparticles. The irregular morphology, with an estimated particle size in the range of ∼30–80 nm and rough surface, demonstrates successful nucleation and formation of ZnCo_2_O_4_ over the conductive carbon framework. It is observed that the incorporation of activated carbon provides a porous framework, facilitating the uniform dispersion of ZnCo_2_O_4_ nanograins and preventing severe particle aggregation. Such a hierarchical porous architecture is highly beneficial for electrochemical applications, as it offers a large specific surface area for redox reactions and short ion diffusion pathways. Corresponding EDX spectrum of ZnCo_2_O_4_/PAC structure, which contain of Zn, Co, C, and O elements as revealed in Fig. 1(e), signifying successful formation of ZnCo_2_O_4_/PAC heterostructure. The resulting EDX spectrum shows characteristic features of the constituent elements, confirming the successful formation of the desired composition. Fig. 1(f) shows the TEM image of ZnCo_2_O_4_/PAC composite, consisting of uniformly distributed ZnCo_2_O_4_ nanocrystallites intimately anchored onto activated carbon framework. Fig. 1(g) illustrates the HRTEM image further confirms the successful formation of the ZnCo_2_O_4_/PAC heterostructure. Distinct lattice fringes with an interplanar spacing of approximately 0.24 nm are assigned to the (311) crystallographic plane of spinel ZnCo_2_O_4_, whereas some amorphous region corresponds to graphitic plane of activated carbon. The coexistence of these well-resolved lattice fringes demonstrates the intimate interfacial contact between crystalline ZnCo_2_O_4_ and the conductive carbon matrix. Such a tightly integrated heterointerface is advantageous for accelerating interfacial charge-transfer kinetics, reducing electron transport resistance, and preserving structural integrity during repeated redox cycling. Fig. 1(i–l) demonstrates the STEM images of ZnCo_2_O_4_/PAC structure and its corresponding elemental mapping of Zn, Co, O and C respectively. These elements further confirm the formation of ZnCo_2_O_4_/PAC structure. In addition, SEM images of ZnCo_2_O_4_ with EDX elemental mapping at both medium and high magnifications are displayed in Fig. S1(a–e). Moreover, SEM images with corresponding EDX of PAC is also illustrated in Fig. S2(a–c).

**Fig. 1:**
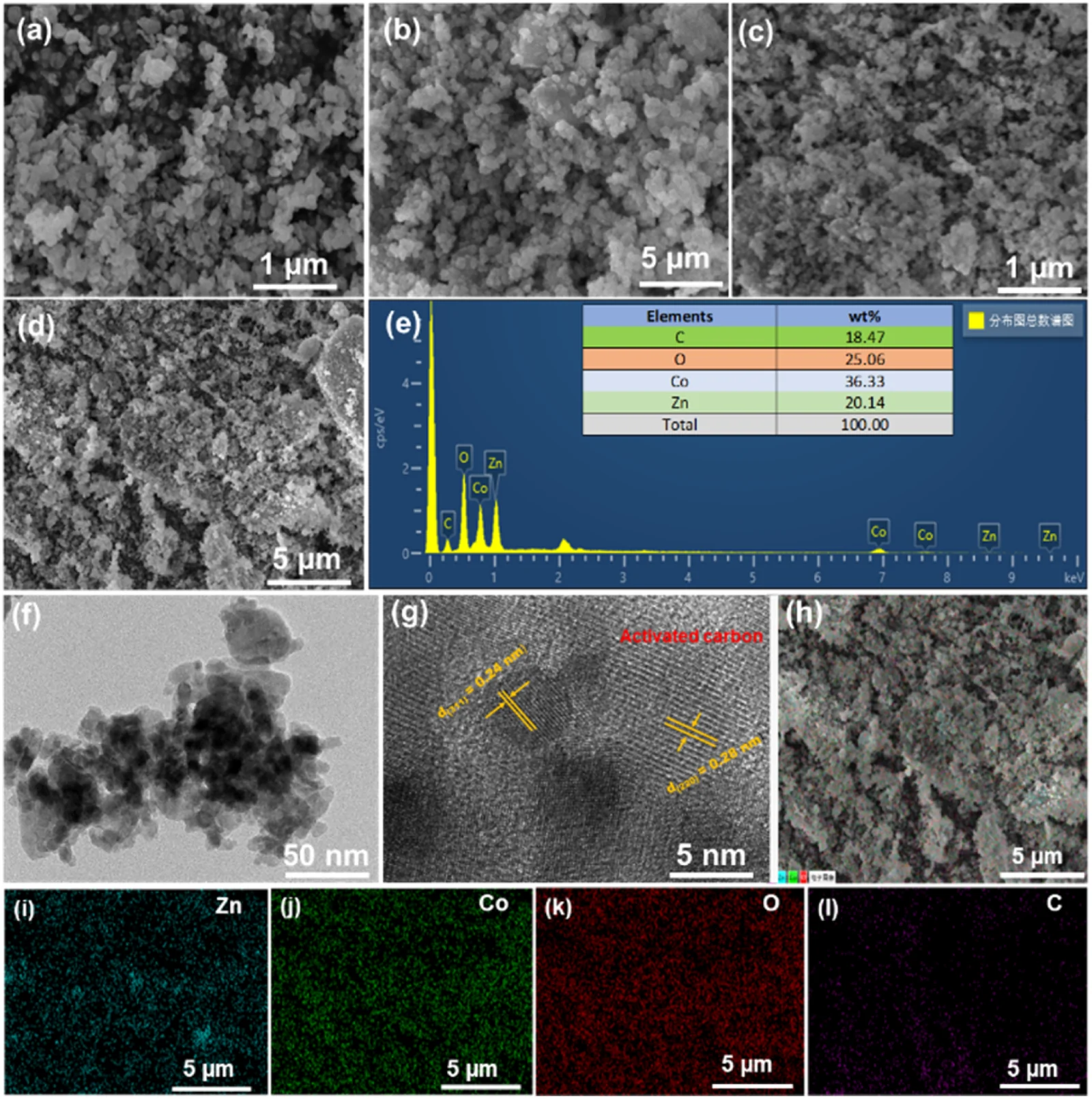
(a and b) SEM images of ZnCo_2_O_4_ (c and d) SEM images of ZnCo_2_O_4_/PAC composite (e) EDX spectrum of ZnCo_2_O_4_/PAC composite (f and g) TEM and HRTEM image of ZnCo_2_O_4_/PAC composite (h–l) STEM and corresponding EDS elemental mapping images of Zn, Co, O and C atoms.

XRD study was accomplished to identify the structural and crystalline phase purity of PAC, ZnCo_2_O_4_ and ZnCo_2_O_4_/PAC samples as depicted in Fig. 2(a). The diffraction pattern of ZnCo_2_O_4_ composed of peaks at 18.6°, 31.4°, 36.5°, 38.4°, 43.8°, 55.2°, 58.8°, and 65.2° resemble to the (111), (220), (311), (222), (400), (422), (511), and (440) planes respectively, were indexed to cubic spinel ZnCo_2_O_4_ structure. The diffraction peaks well matched with the standard XRD pattern, marked with (JCPDS no. 23-1390).^20^ Sharp, well-defined peaks in the pattern imply that the composite has a maximum level of crystallinity. Additionally, a broad diffraction hump centred at 26° is attributed to PAC associated to the (002) plane, which is feature of amorphous C. Importantly, a slight shift towards a smaller 2*θ* value in the ZnCo_2_O_4_/PAC composite is noticed, indicating that ZnCo_2_O_4_ structure expands by the addition of PAC. The XRD analysis shows the successful synthesis of PAC, ZnCo_2_O_4_ and ZnCo_2_O_4_/PAC composite, showing good phase purity and excellent crystallinity, making it good candidate for charge storage mechanism in supercapacitors.

**Fig. 2:**
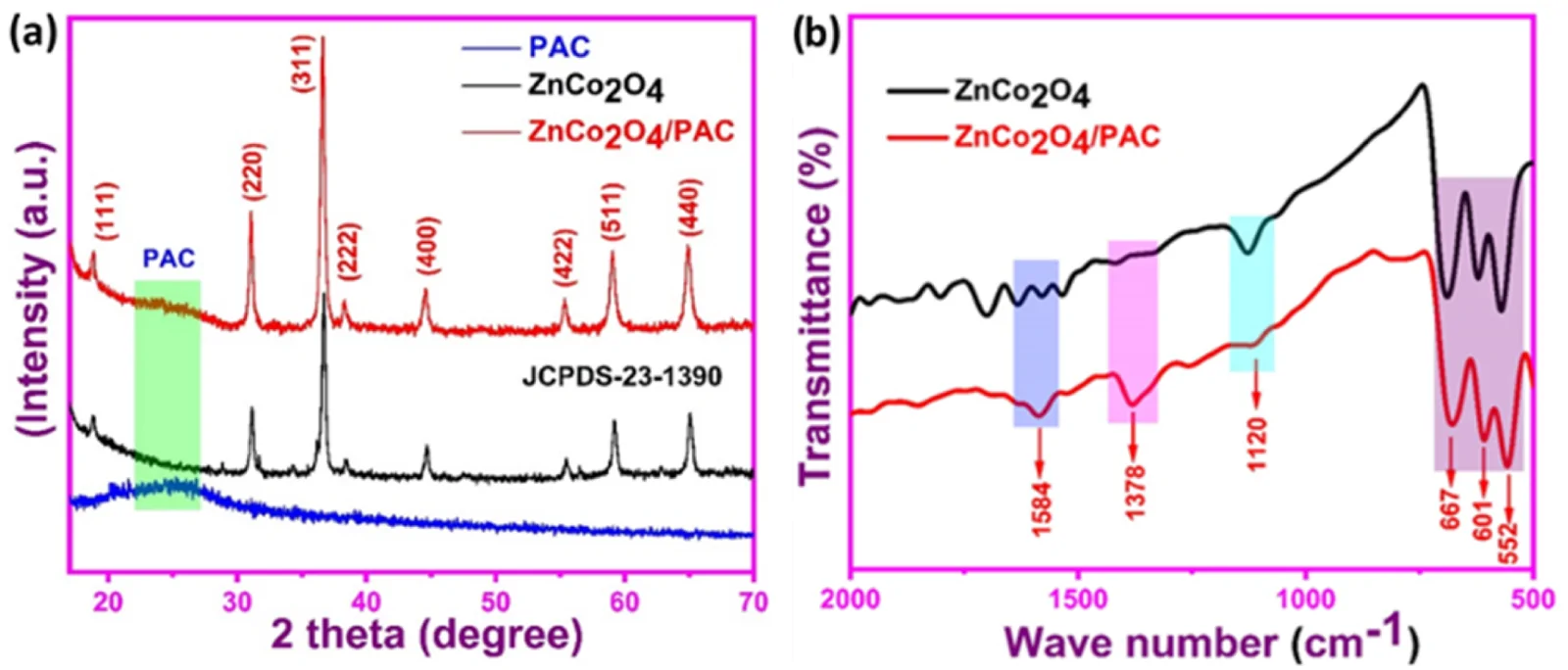
(a) XRD pattern of PAC, ZnCo_2_O_4_ and ZnCo_2_O_4_/PAC composite (b) FTIR analysis of ZnCo_2_O_4_ and ZnCo_2_O_4_/PAC composite.

To investigate the functional groups and bonding features in the synthesized materials (ZnCo_2_O_4_, ZnCo_2_O_4_/PAC), FTIR spectroscopy was done over the wavenumber range of 2000–500 cm^−1^, as shown in Fig. 2(b). The ZnCo_2_O_4_/PAC spectrum shows distinct peaks located at 1584, 1378, 1120, 667, 601 and 552 cm^−1^. The band at 1584 cm^−1^ which can be ascribed to the C–C stretching vibrations of aromatic carbon structure, confirming the presence of activated carbon. The peak at 1378 cm^−1^ corresponds to C–H bending vibrations, while the peak at 1120 cm^−1^ is attributed to C–O stretching, demonstrating hydroxyl or ether groups on the carbon surface. Additionally, the absorption peaks at low frequency region, 667 cm^−1^, 601 cm^−1^ and 552 cm^−1^ are assigned to metal–oxide (Zn–O and Co–O) bonds confirming the formation of spinel structure of both samples. Interestingly, compared to pristine ZnCo_2_O_4_, the ZnCo_2_O_4_/PAC composite shows additional peaks related to carbon functional groups and there is a slight shift in the ZnCo_2_O_4_/PAC structure towards a lower wave number. This change suggests a interfacial interaction between ZnCo_2_O_4_ and the PAC framework.

The nitrogen adsorption–desorption isotherms of pristine ZnCo_2_O_4_ and the ZnCo_2_O_4_/PAC composite are shown in Fig. S3(a and b). Both samples show type-IV adsorption–desorption isotherms with a distinct hysteresis loop at intermediate-to-high relative pressures (*P*/*P*_0_). The adsorption behaviour confirms the coexistence of mesopores and interparticle macropores. The BET analysis reveals that the pristine ZnCo_2_O_4_ possesses a specific surface area of 10.81 m^2^ g^−1^, whereas the ZnCo_2_O_4_/PAC composite exhibits a significantly enhanced surface area of 14.41 m^2^ g^−1^. This noticeable increase reveals the effective contribution of activated carbon in generating additional accessible surface sites and preventing particle agglomeration. The interconnected porous carbon framework not only increases the exposed electrochemically active interface but also facilitates efficient electrolyte penetration.

To explore the functionalization of surfaces and chemical valence states in ZnCo_2_O_4_/PAC composite, XPS measurements were done. Fig. 3(a) shows the high-quality spectrum of Zn 2p peaks indicates a stable chemical composition for Zn within the hybrid structure. The oxidation state of Zn is divalent and demonstrated by the spectrum's two prominent peaks at 1023.5 and 1046.7 eV, which correspond to the electronic states of Zn 2p_3/2_ and Zn 2p_1/2_, respectively. Remarkably, the binding energy separation between these two peaks is 23.2 eV.^21^ The absence of satellite peaks in the spectra indicates that zinc does not exist in many oxidation states. The Co 2p deconvoluted spectrum in Fig. 3(b) has two peaks roughly at 784.6 and 799.1 eV, which are similar to the two satellite peaks of Co 2p_3/2_ and Co 2p_1/2_. The deconvoluted peaks at the binding energies of 784.2 and 799.5 eV are attributed to Co^3+^, whereas the peaks at 786.3 and 801.5 eV are attributed to Co^2+^.^22,23^ The high-resolution spectrum of C is as shown in Fig. 3(c). There are three peaks of carbon are acknowledged, the major peak at ∼283.9 eV ascribed to C–C/C=C bonds corresponding to graphitic carbon from activated carbon, peak at ∼284.8 eV indicating the presence of hydroxyl group (C–O). Other peak at ∼285.5 corresponding to O–C=O functional groups. The existence of these peaks demonstrates the formation of ZnCo_2_O_4_/PAC hybrid structure. The presence of these functional groups is important for improving the performance as well as improve the electrode's cyclic stability. The O 1s spectrum is seen in Fig. 3(d) and is split into two peaks: the oxygen vacancy at 531.2 eV and the lattice oxygen of the metal–oxygen bonds at 529.6 eV defects provide plentiful active sites, which are crucial for high performance supercapacitor applications. For comparative study XPS measurements of pristine ZnCo_2_O_4_ sample also done, as shown in Fig. S4. It is observed that, the incorporation of PAC induces a systematic shift of the Zn 2p, Co 2p, and O 1s core-level spectra toward higher binding energies relative to pristine ZnCo_2_O_4_. This positive shift signifies a pronounced interfacial electronic interaction between ZnCo_2_O_4_ and the conductive PAC framework, resulting in charge redistribution and interfacial electronic interaction at the heterointerface. The increase in binding energy suggests a partial decrease in the electron density surrounding the Zn, Co, and O atoms. Such interfacial charge modulation optimizes the electronic structure of the composite, facilitating more efficient charge separation and accelerating electron-transfer kinetics during electrochemical reactions.

**Fig. 3:**
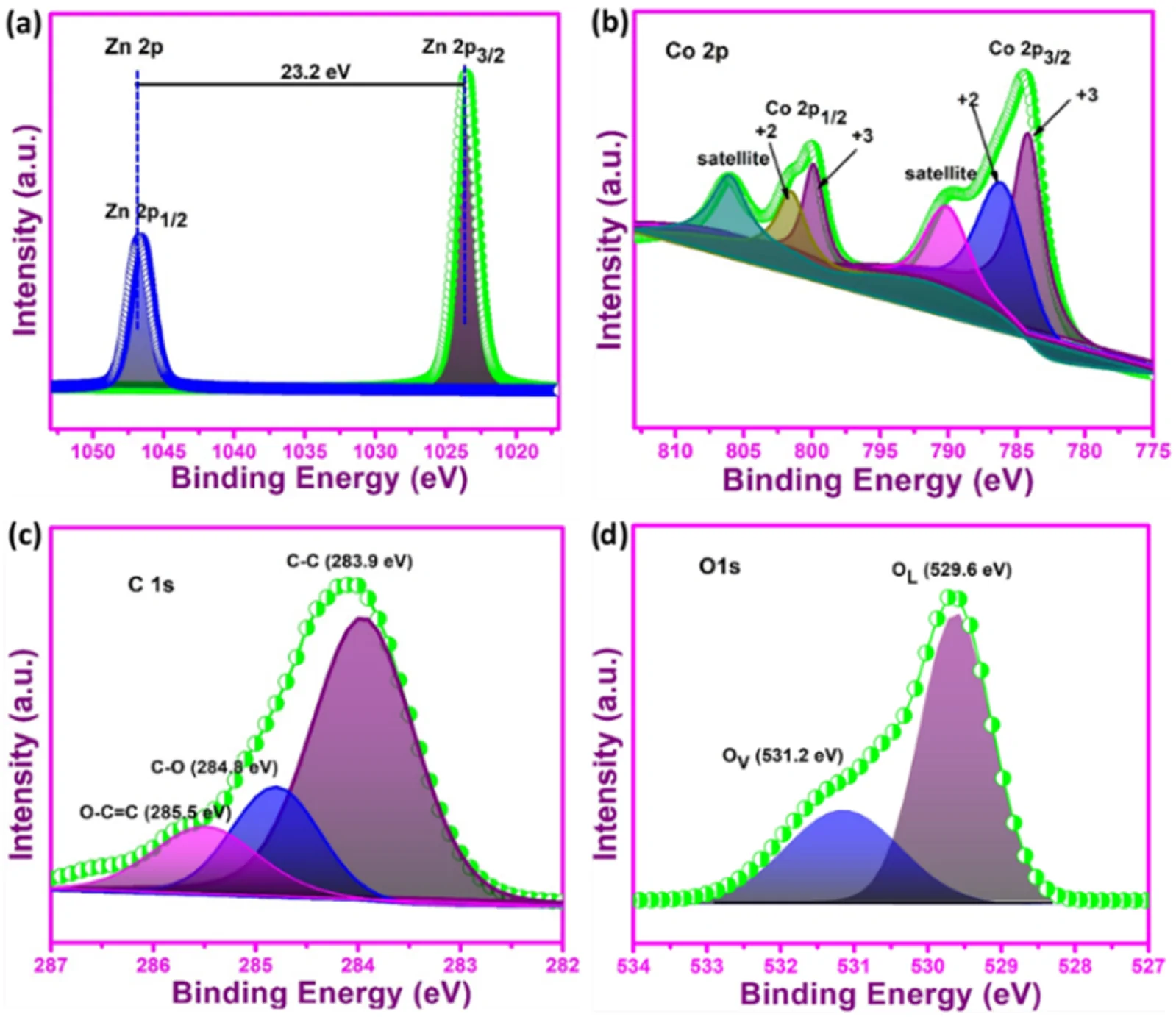
(a–d) Shows the high-resolution spectra of Zn 2p, Co 2p, C 1s, and O 1s, respectively.

## 4. Electrochemical performance

The electrochemical responses of ZnCo_2_O_4_ and ZnCo_2_O_4_/PAC electrodes were evaluated. Fig. 4(a) demonstrates the comparative CV behaviour of ZnCo_2_O_4_ and ZnCo_2_O_4_/PAC electrodes at a scan rate of 60 mV s^−1^. Compared to other electrodes, the ZnCo_2_O_4_/PAC electrode has larger CV area, which suggests higher charge-storage capability. The presence of clearly defined oxidation-reduction peaks suggests that reversible redox processes are primarily responsible for charge storage. The enhanced response can be attributed to the synergistic interaction of ZnCo_2_O_4_ and PAC with high surface area. The response of ZnCo_2_O_4_/PAC electrode at scan rates of 10 to 60 mV s^−1^ are presented in Fig. 4(b). The presence of well-defined anodic and cathodic redox peaks confirms the pseudocapacitive charge-storage behaviour of the electrode. The exceptional electrochemical kinetics, reversibility, and high-rate capability are demonstrated by the CV curves, which retain their overall shape even at the high scan rate. The impact of capacitive effects and diffusion kinetics on the electrode's electrochemical performance is highlighted by the specific capacitance.

**Fig. 4:**
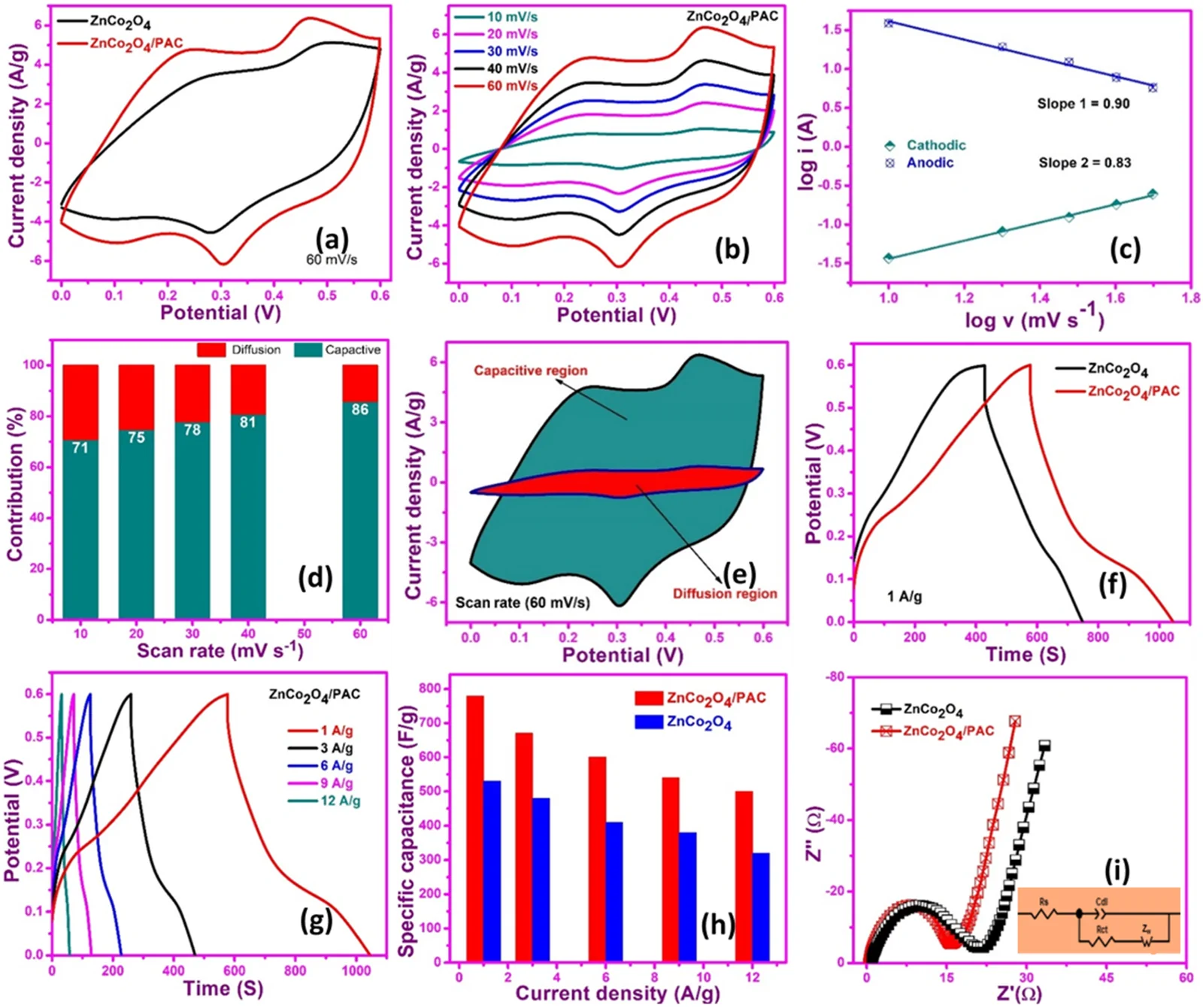
(a) CV profiles comparing of ZnCo_2_O_4_ and ZnCo_2_O_4_/PAC electrodes at recorded at 60 mV s^−1^ (b) CV curves of ZnCo_2_O_4_/PAC electrode collected over a scan rate range from 10 to 60 mV s^−1^ (c) logarithmic relationship between scan rate (log *v*) and peak current (log *i*), demonstrating the charge storage kinetics (d) diffusion *versus* capacitive-controlled charge storage behaviour (e) percentage of capacitive and diffusion capacitance contributions of ZnCo_2_O_4_/PAC electrode at 60 mV s^−1^ (f) comparative analysis of GCD curves of ZnCo_2_O_4_ and ZnCo_2_O_4_/PAC electrodes at 1 A g^−1^ (g) GCD curves of ZnCo_2_O_4_/PAC electrode at current density of 1 to 12 A g^−1^ (h) the calculated specific capacitance values corresponding to all electrodes (i) Nyquist plot of the all electrodes (inset shows circuit diagram).

The possible oxidation reduction electrochemical reactions in KOH electrolyte solution are based on following equations;^24^ZnCo_2_O_4_ + H_2_O + OH^−^ ↔ 2CoOOH + ZnOOH + e^−^ZnOOH + OH^−^ ↔ ZnO_2_ + H_2_O + e^−^CoO_2_ + 2H_2_O + e^−^ ↔ Co(OH)_2_ + 2OH^−^

To further elucidate the charge-storage kinetics, Dunn's method was employed based on the power-law peak current on scan rate. It was used to quantitatively resolve the relative contributions of surface-controlled capacitive processes and diffusion-controlled faradaic reactions in the electrode;^25^

4 *i*_peak_ = *av*^*b*^ or log(*i*_peak_) = *b* log(*v*) + log (*a*)

where ‘*a*’ is constant parameter and ‘*b*’ characterizes the slope (eqn (4)). The obtained *b* values for peaks α and β are 0.83 and 0.90, respectively (Fig. 4(b)). The *b* values approaching unity imply improved capacitive kinetics of the ZnCo_2_O_4_ electrode. Fig. 4(c) depicts the linear relationship between log *v* and log *i*, revealing a hybrid charge-storage mechanism. Moreover, the ratio of capacitive and diffusion contributions is observed. As reported in the literature, the current response at a fixed potential, *i*(*v*), can be de-convoluted into a surface-controlled capacitive term (*k*_1_*v*) and a diffusion-limited faradaic term (*k*_2_*v*^1/2^). The grouping of charge-storage pathway is carried out following the equation below;^26^

5 *i*(*v*) = *k*_1_*v* + *k*_2_*v*^1/2^

To simplify the quantitative analysis of the individual charge-storage contributions, eqn (5) was reformulated into a linear form;6
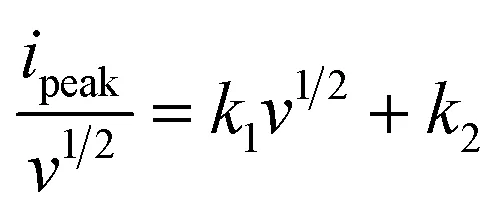


The capacitive contribution increased expressively from 71% to 86% as from low scan rate (10 mV s^−1^) to high scan rate (60 mV s^−1^) as depicted in Fig. 4(d). Fig. 4(e) shows the relative contributions of the surface-controlled capacitive and diffusion-controlled charge-storage mechanisms 60 mV s^−1^. The graph illustrates that the diffusion contribution rate is 14% (red colour) and the capacitive contribution rate is 86% (zinc colour). At lower scan rate (10 mV s^−1^), the prolonged interaction between electrochemically active sites of electrode and electrolyte ions promotes diffusion-limited faradaic reactions. In contrast, at a scan rate of 60 mV s^−1^, the capacitive contribution predominates because the limited timescale available for ion diffusion suppresses bulk diffusion-controlled reactions, favoring rapid surface faradaic processes. Furthermore, the interconnected porous architecture of the ZnCo_2_O_4_/PAC electrode minimizes internal resistance, improves electrolyte accessibility, and facilitates rapid ion diffusion and charge-transfer kinetics during repeated charging/discharging processes. These results advocate that ZnCo_2_O_4_/PAC electrode is a highly attractive and high-performing electrode for electrochemical system. In addition, the CV response of pristine ZnCo_2_O_4_ electrode is illustrated in Fig. S5(a).

GCD measurements were also carried out to examine the charge storage performance. The comparative GCD curve of ZnCo_2_O_4_ and ZnCo_2_O_4_/PAC electrodes at 1 A g^−1^ is shown in Fig. 4(f). The ZnCo_2_O_4_/PAC electrode exhibits a longer discharge time than the pristine ZnCo_2_O_4_ electrode, Fig. 4(g) demonstrating its higher specific capacitance. The calculated capacitance is about 781, 670, 600, 550 and 520 F g^−1^ at 1, 3, 6, 9, and 12 A g^−1^, respectively. It is interesting to note that, the capacitance of ZnCo_2_O_4_/PAC electrode is better than pristine ZnCo_2_O_4_ (530 F g^−1^) electrode at 1 A g^−1^ as shown in Fig. 4(h). The longer charge–discharge duration observed at 1 A g^−1^ indicates a higher specific capacitance, because the synergistic effect of both ZnCo_2_O_4_ and PAC with more active sites and high surface area. These remarkable results highlight the need for additional investigation into improving the material's electron transport and ion accessibility for better electrochemical performance in a range of operating conditions. Clearly, the ZnCo_2_O_4_/PAC electrode has a better performance as compared to the pristine ZnCo_2_O_4_ and reported work, as shown in Fig. S6 and Table 1. Furthermore, the GCD behaviour of pristine ZnCo_2_O_4_ electrode is shown in Fig. S5(b).

**Table 1:** Comparative analysis of the electrochemical performance of ZnCo_2_O_4_, activated carbon, and the present electrode

Electrodes	Specific capacitance (F g^−1^)	Current density (A g^−1^)	Energy density Wh kg^−1^	Cyclic stability (%)	Cycle numbers	Reference
ZnCo_2_O_4_@N-GO/PANi	720	1.5	—	96.4	10 000	30
ZnCo_2_O_4_	593	1	—	—	—	31
RGO/ZnCo_2_O_4_	755.38	1	10.75	93.47	2000	32
ZnCo_2_O_4_	680	1	—	90	2000	33
ZnCo_2_O_4_ nanorods	315	2	25.45	77	3000	20
Pecan shell-derived activated carbon	269	2	2.1	—	15 000	34
ZnCo_2_O_4_/AC	692	1	221	88	5000	35
Activated carbon (AC)	195	0.5	—	76	50 000	36
ZnCo_2_O_4_	331.2	2	38.1	—	7000	37
ZnCo_2_O_4_	422	1	29.8	83	10 000	38
**ZnCo**_**2**_ **O4/PAC**	**781**	**1**	**50**	**92**	**6000**	**Our work**

The Nyquist plots of ZnCo_2_O_4_ and ZnCo_2_O_4_/PAC electrodes are shown in Fig. 4(i). It can be observed that the ZnCo_2_O_4_/PAC electrode displays the smallest semicircle diameter, reflecting a lower *R*_ct_ compared with ZnCo_2_O_4_ electrode. This implies that the hybrid composition facilitates electron conduction and rapid charge transfer, owing to its larg surface area. The fitted parameters are summarized in Table S1. ZnCo_2_O_4_ and ZnCo_2_O_4_/PAC electrodes exhibited calculated *R*_s_/*R*_ct_ values of 1.6/19.5 Ω and 1.2/14.2 Ω, respectively. In comparison to the ZnCo_2_O_4_ electrode, the ZnCo_2_O_4_/PAC electrode exhibits lower *R*_s_ and *R*_ct_ values, suggesting fast charge-transfer kinetics and lower internal resistance. The remarkably reduced *R*_ct_ value indicates rapid interfacial charge transfer, enhanced electrical conductivity and efficient ion diffusion.

### 4.1 The performance of ZnCo_2_O_4_/PAC//AC asymmetrical supercapacitor device

For real-world application, an asymmetric supercapacitor (ZnCo_2_O_4_/PAC//AC) device was designed to evaluate the performance of ZnCo_2_O_4_/PAC and activated carbon (AC) electrodes. The fabricated device was assembled using ZnCo_2_O_4_/PAC as the positive electrode and activated carbon (AC) as the negative electrode, with 1.0 M KOH serving as the electrolyte. Fig. 5(a) illustrates the schematic of assembled ZnCo_2_O_4_/PAC//AC device. The CV curves of the ASC device recorded at scan rates ranging from 10 to 60 mV s^−1^ within a potential window of 0–1.5 V (Fig. 5(b)) retain nearly identical shapes as the scan rate increases, demonstrating good capacitive reversibility and rapid charge-transport kinetics. Fig. 5(c) presents the GCD profiles of the ZnCo_2_O_4_/PAC//AC device recorded at numerous current densities. As established in Fig. 5(d), the device attains a capacitance of 160 F g^−1^ at 1 A g^−1^ and retained 61% of its capacitance at 12 A g^−1^ (inset illustrates the optical image of the developed device). The Ragone plot illustrated in Fig. 5(e) reveals that the ASC device attains an excellent balance between energy density and power density, outperforming many previously reported pristine ZnCo_2_O_4_ electrodes, such as ZnCo_2_O_4_@MnMoO_4_ (48 W kg^−1^ and 695 Wh kg^−1^),^20^ ZnCo_2_O_4_@NiO//rGO (46 Wh kg^−1^ and 800 W kg^−1^),^27^ N-PAC//PVA-KOH//Co-PAC (45 Wh kg^−1^ at 846 W kg^−1^),^28^ ZnCo_2_O_4_ (28.6 Wh kg^−1^ at 100 W kg^−1^).^29^Fig. 5(f) shows the long-term cyclic stability of the ASC device, which retains approximately 92% of its initial capacitance after 6000 charge–discharge cycles, indicating excellent electrochemical stability. Furthermore, the inset highlights the highly reproducible charge–discharge behavior observed during the initial 10 cycles at a current density of 6 A g^−1^.

**Fig. 5:**
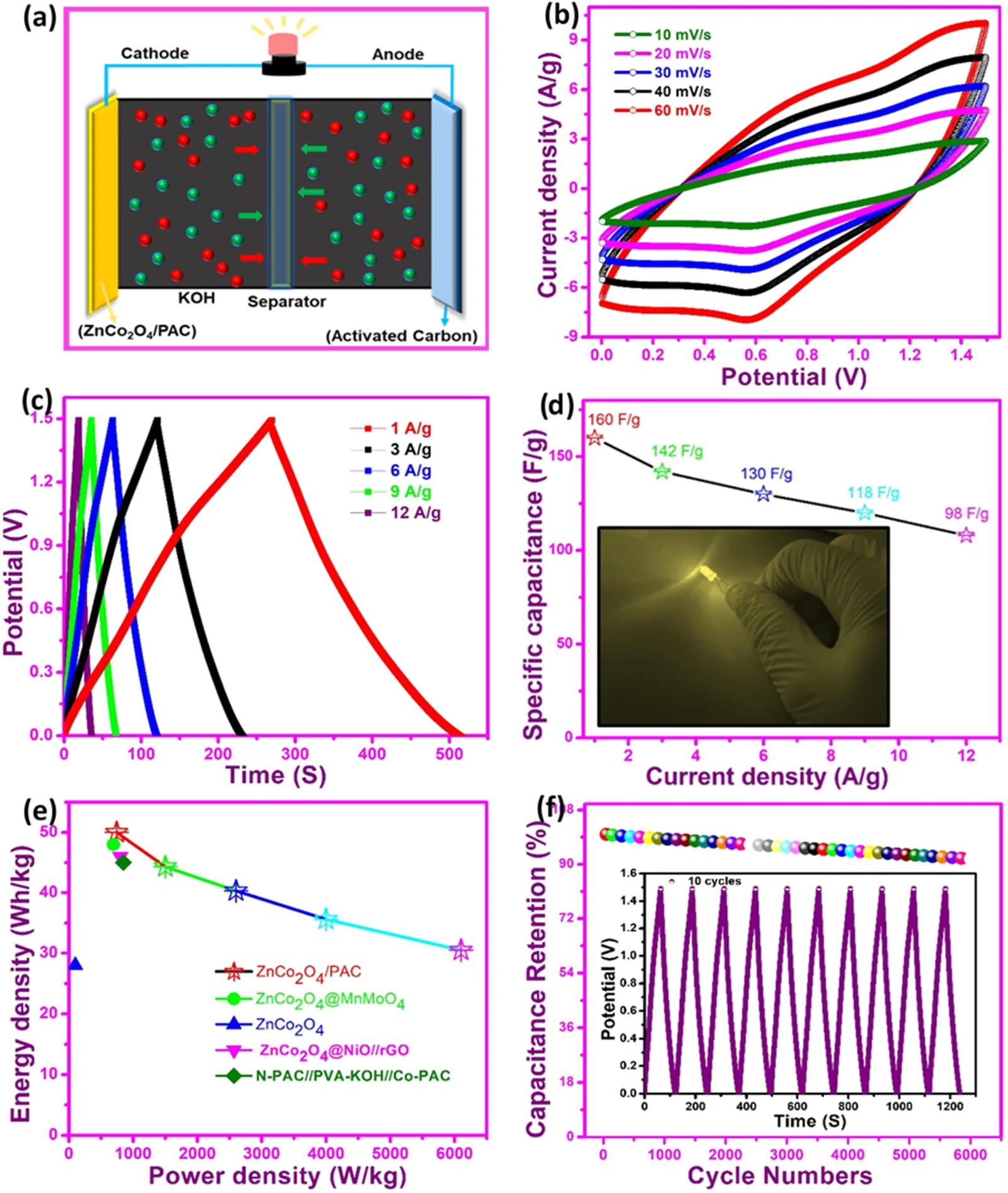
Electrochemical evaluation of the of ZnCo_2_O_4_/PAC//AC device (a) schematic design of fabricated device (b) CV response at scan rates of 10 to 60 mV s^−1^ of the device (c) GCD profiles obtained at various current densities (d) calculated capacitance values derived from the GCD curves (the inset illustrates the optical image of the developed device) (e) Ragone plot signifying the relationship between energy density and power density (f) cycling stability performance of the device (inset shows the charge–discharge curves at the first 10 cycles).

## 5. Conclusion

In summary, the ZnCo_2_O_4_/PAC nanocomposite was successfully synthesized *via* a facile and cost-effective hydrothermal method. The synergistic combination of ZnCo_2_O_4_ with porous activated carbon significantly enhanced the electrochemical performance. The fabricated nanocomposite exhibited outstanding capacitive performance, excellent rate capability, and remarkable cycling stability, highlighting its strong potential as a high-performance electrode material for supercapacitor applications. The fabricated electrode presented a remarkable specific capacitance of 781 F g^−1^ as compared with the pristine ZnCo_2_O_4_ electrode at 1 A g^−1^. Additionally, the ZnCo_2_O_4_/PAC//AC asymmetric supercapacitor device demonstrated outstanding cyclic stability with 92% capacitance retention after 6000 cycles. Moreover, the device achieved a high energy density of 50 Wh kg^−1^ at a power density of 743.8 W kg^−1^. The enhanced electrochemical performance can be attributed to the synergistic effect of the integrated components, which promotes electron transport and preserves structural integrity by reducing stress accumulation during repeated charge–discharge cycles. These findings demonstrate that the ZnCo_2_O_4_/PAC nanocomposite is a promising electrode material for designing high-performance supercapacitors.

## Author contributions

The author, J. S. L., W. A., and C. L. conceived the idea. W. A. and C. L. synthesized the material. W. A., M. E. M., C. D., and M. N. K., analyzed the data. Authors S. A., M. U. Y., R. B., and A. contributed to the preparation of the initial draft and participated in revising and improving the manuscript through to its final version. The manuscript was prepared through the contributions of all authors. All authors reviewed and approved the final manuscript.

## Conflicts of interest

No financial conflicts of interest are declared by the authors.

## Supplementary Material

RA-016-D6RA04675D-s001

## Data Availability

All related data are available from the authors upon request. Supplementary information (SI) is available. See DOI: https://doi.org/10.1039/d6ra04675d.
