# Supplementary material for: Peanut shell-derived activated carbon incorporated with ZnCo2O4 in alkaline solution for high performance asymmetric supercapacitors
Source: RSC Adv. 2026 Sep 3;16(43):52735–43. doi: 10.1039/d6ra04675d (PMC13539495; doi:10.1039/d6ra04675d)
Supplement: Supplementary file 1 [file RA-016-D6RA04675D-s001.pdf]

Supplementary Information (SI) for RSC Advances.
This journal is © The Royal Society of Chemistry 2026

Supplementary information

Peanut shell-derived activated carbon incorporated with $ZnCo_2O_4$ in alkaline solution for high performance asymmetric supercapacitors

Jing-Song Lv[a], Lei-Cong[a], Waseem Abbas[b], Rehana Bibi[c], Changlin Dong[d], Muhammad Ehsan Mazhar[b], Imran Shakir[e], M. Naziruddin Khan[e], Shakeel Abbas[f], Muhammad Usman Yousaf[b], Areeba[b]

[a]Analytical and Testing Center, Guizhou University of Engineering Science, Guizhou, 551700, China
[b]Institute of Physics, Bahauddin Zakriya University, Multan, 60800, Pakistan
[c]Department of Chemistry, Women University, Sawabi, Pakistan
[d]School of Material Science and Engineering, Shanghai Jiao Tong University, Shanghai 200240, China
[e]Department of Physics, Faculty of Science, Islamic University of Madinah, Madinah 42351, Saudi Arabia
[f]Department of Chemistry, Government College University, Faisalabad, Pakistan.

Correspondence: Waseem Abbas; Dr.waseemabbas@bzu.edu.pk

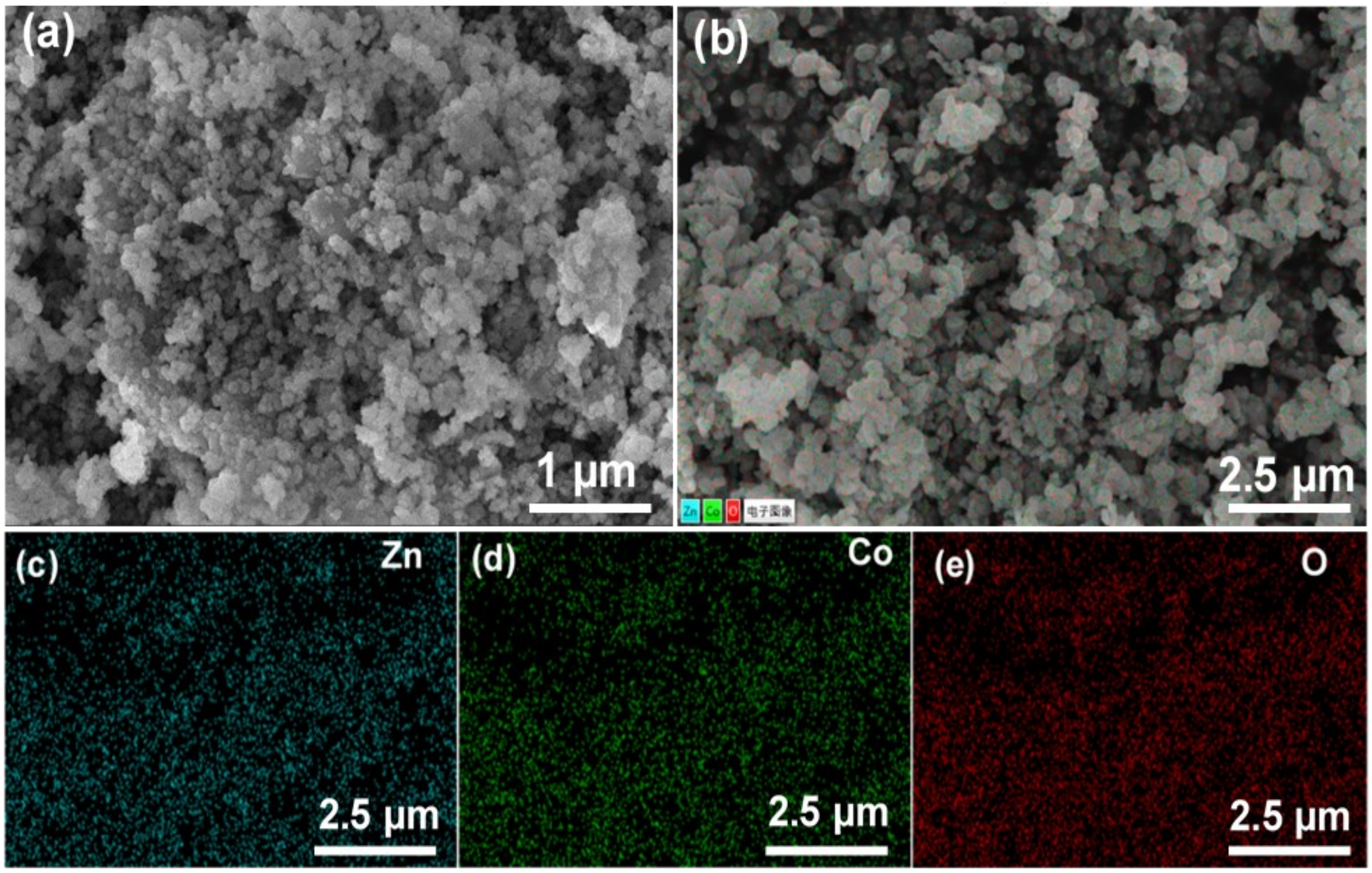

Figure. S1. (a, b) SEM images of $ZnCo_2O_4$ structure at different magnifications (c-e) elemental mapping of Zn, Co and O.

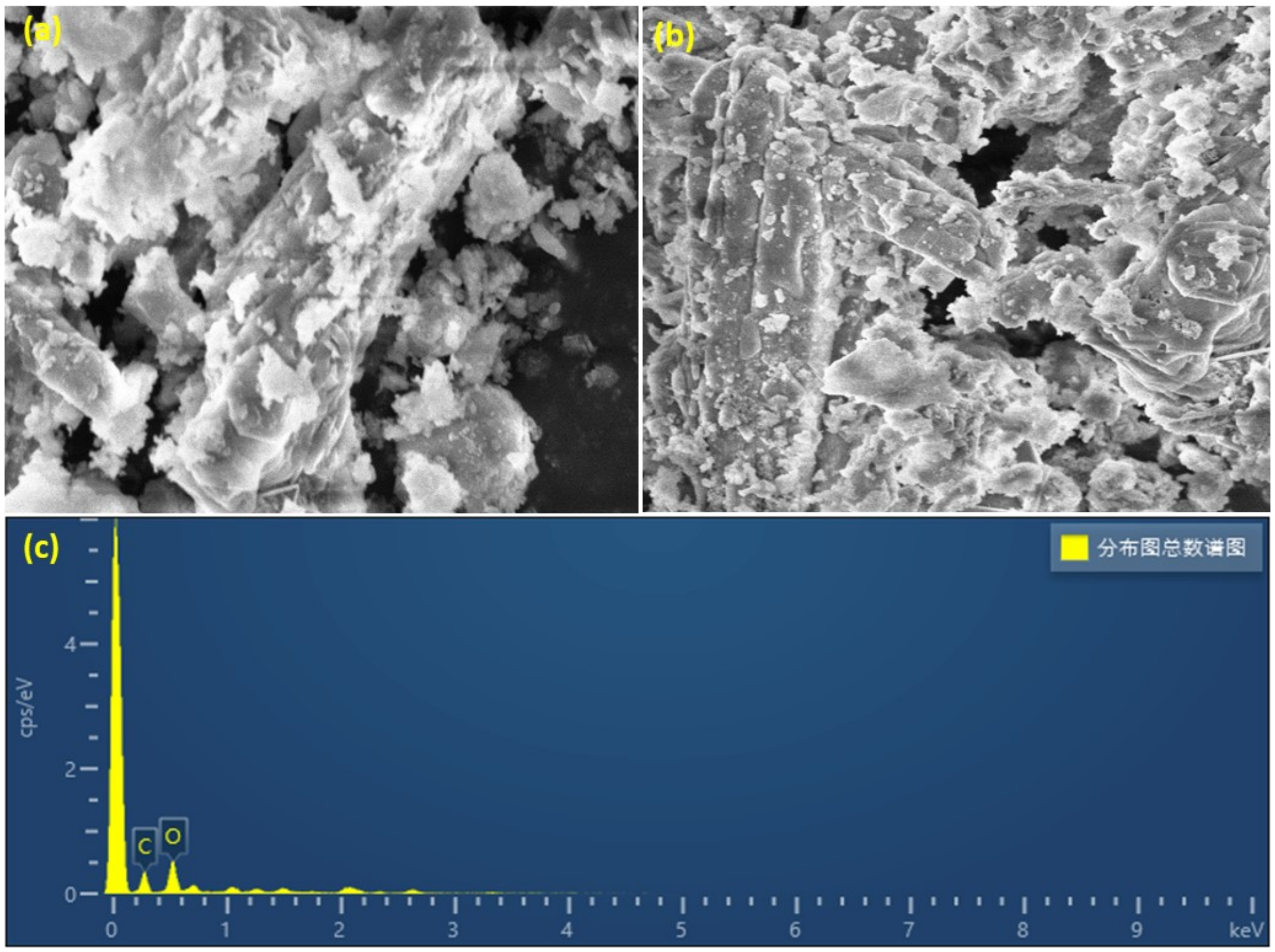

Figure. S2. (a, b) SEM images of activated carbon (c) corresponding EDX.

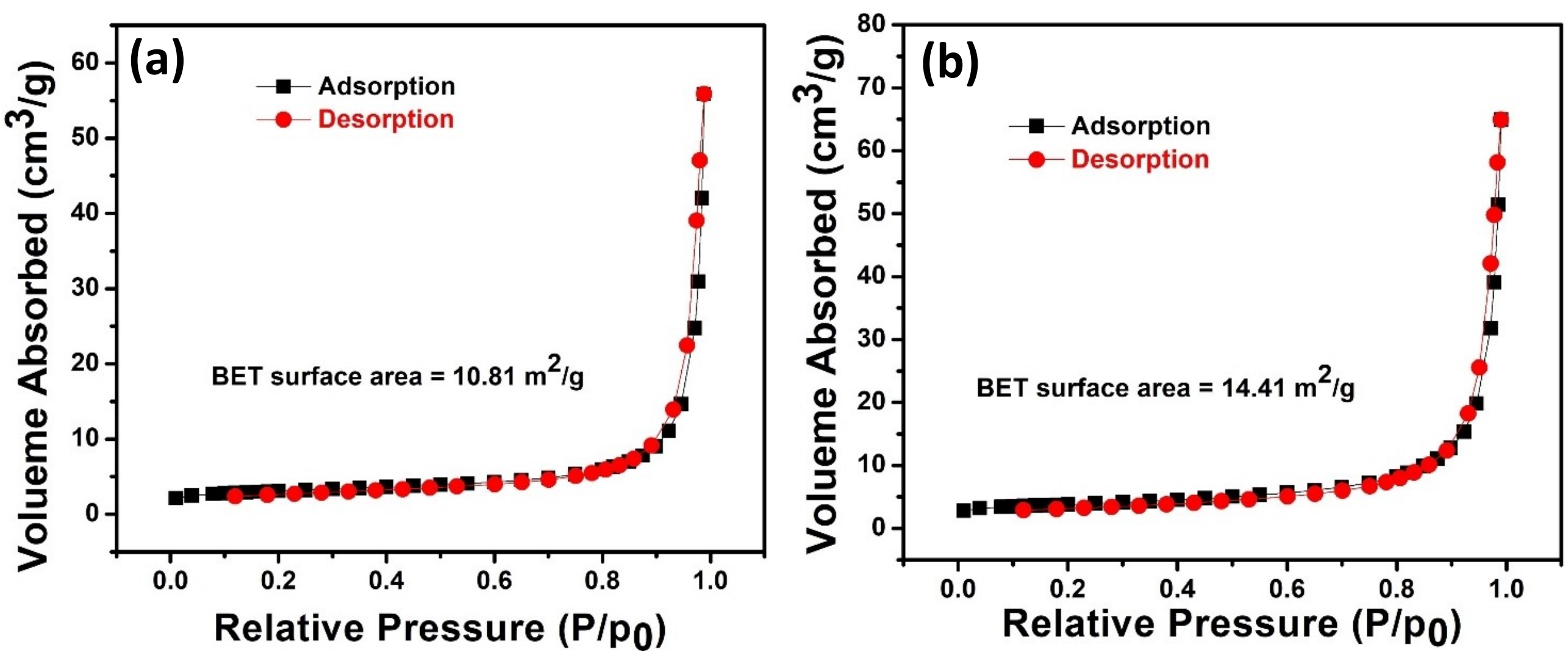

Figure. S3. (a, b) Nitrogen absorption-desorption isotherm of $ZnCo_2O_4$ and $ZnCo_2O_4$/PAC composite.

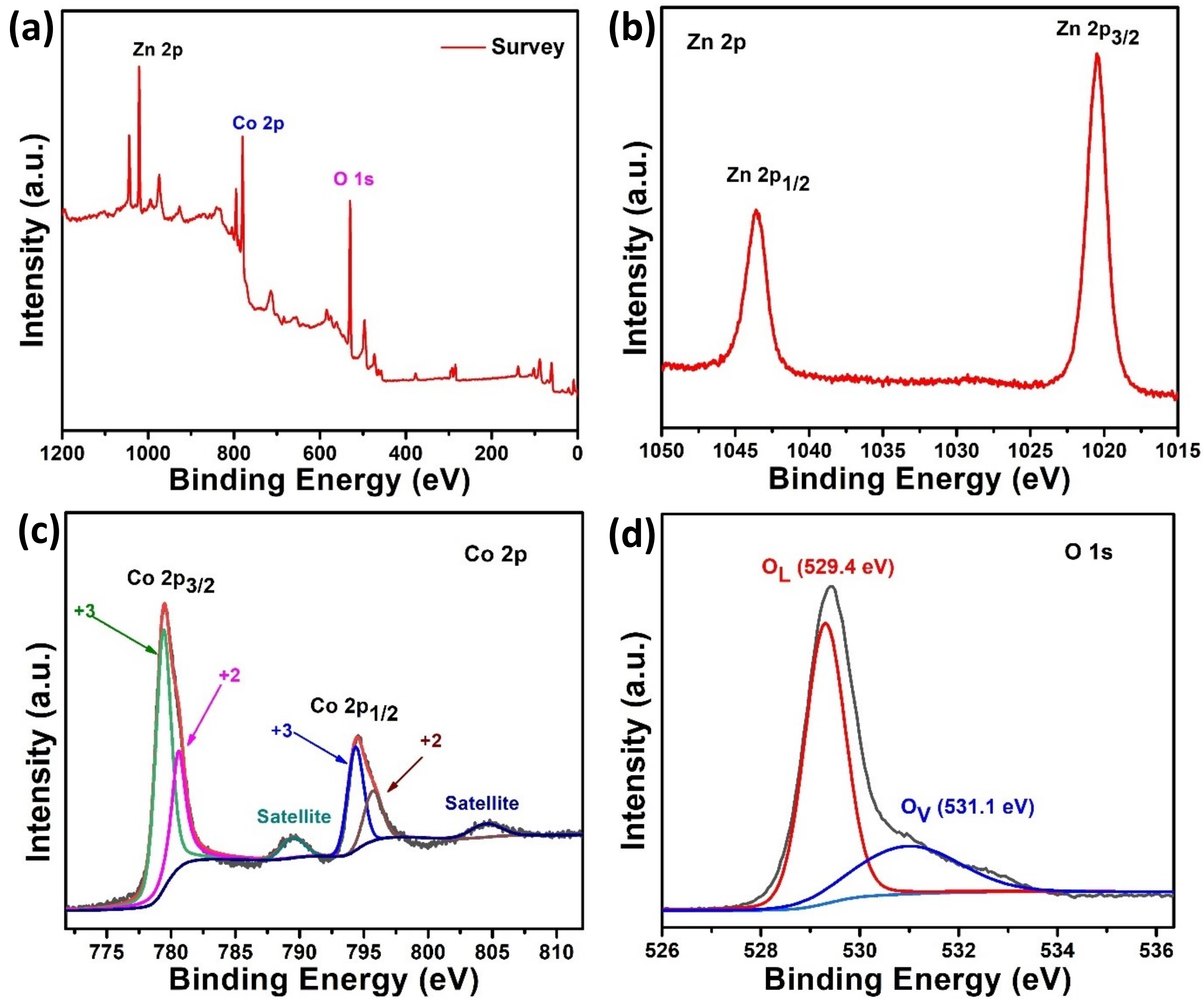

Figure. S4. (a) XPS survey spectra of $ZnCo_2O_4$ (b–c) high-resolution spectra of Zn 2p, Co 2p, and O 1s, respectively.

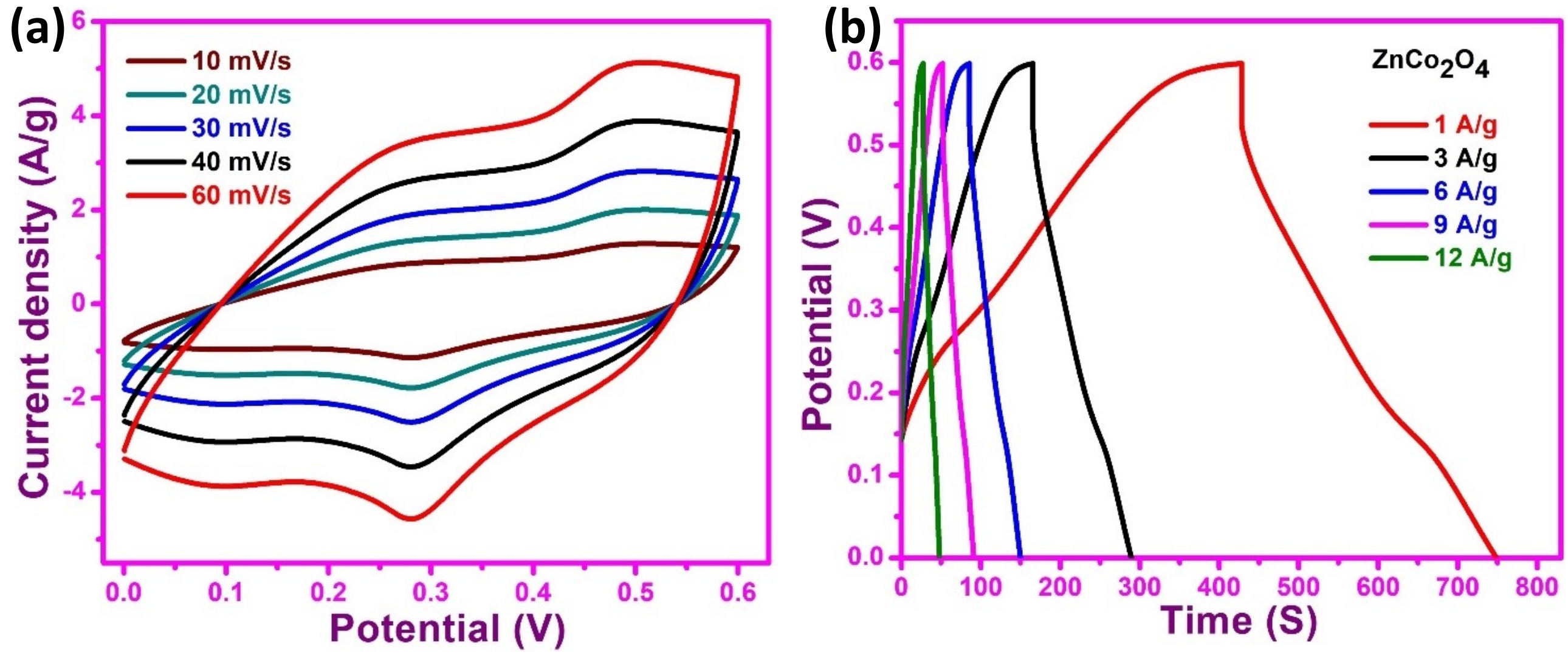

Figure. S5. (a) CV response of $ZnCo_2O_4$ electrode at scanning rate at scanning rate of 10 to 60 mV/s (b) GCD behavior of $ZnCo_2O_4$ electrode at current density of 1 to 12 A/g.

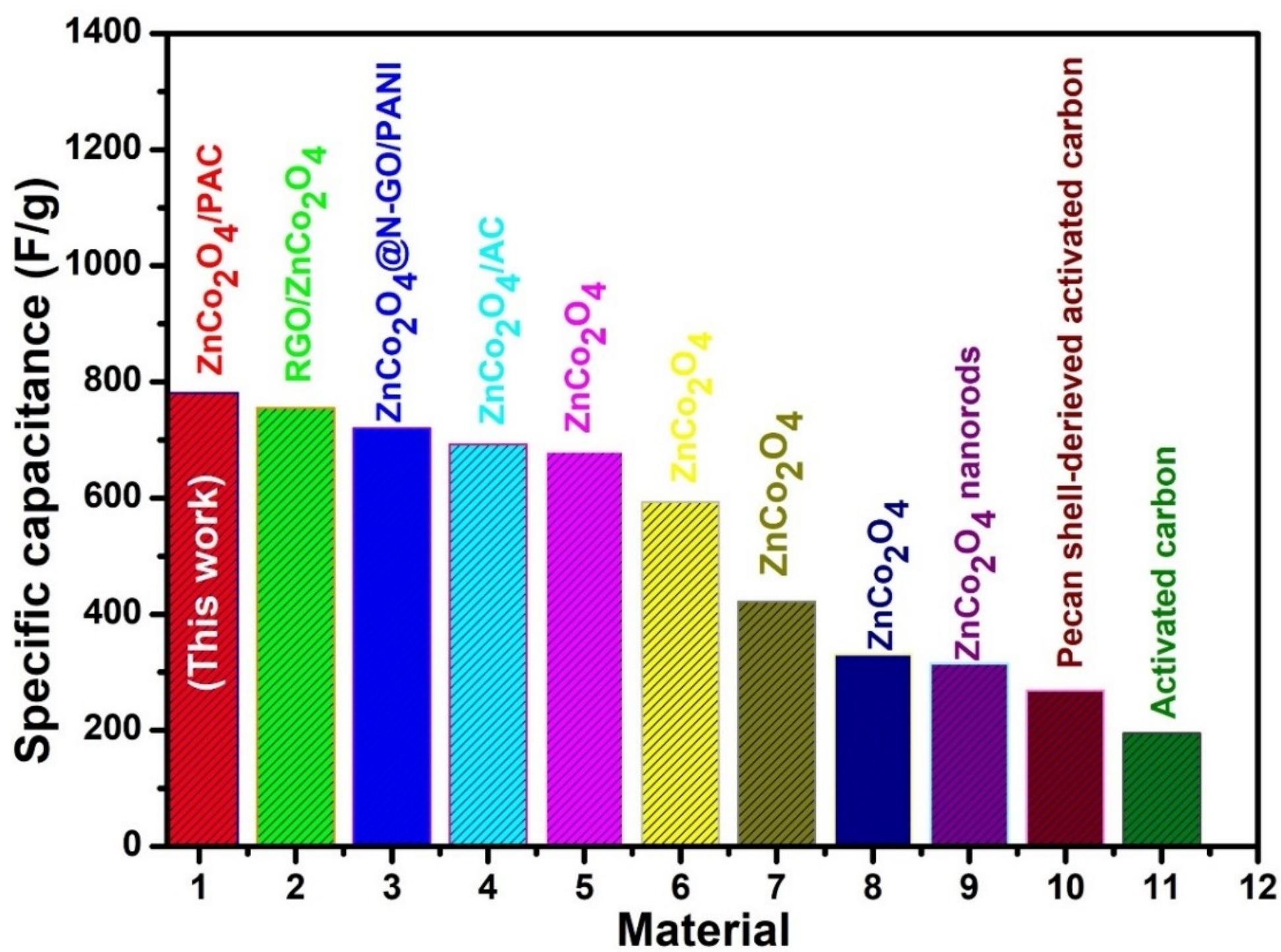

Figure. S6. Comparative analysis of specific capacitance between previously reported $ZnCo_2O_4$-based study and current research study ($ZnCo_2O_4$/PAC).

Table. S1. Comparative analysis of kinetic parameters of $ZnCo_2O_4$ and $ZnCo_2O_4$/PAC electrodes.

Material	Rs (Ω)	Rct (Ω)
$ZnCo_2O_4$	1.6	19.5
$ZnCo_2O_4$/PAC	1.2	14.2
